# Reversible Data Hiding Based on DNA Computing

**DOI:** 10.1155/2017/7276084

**Published:** 2017-02-08

**Authors:** Bin Wang, Yingjie Xie, Shihua Zhou, Changjun Zhou, Xuedong Zheng

**Affiliations:** ^1^Key Laboratory of Advanced Design and Intelligent Computing, Dalian University, Ministry of Education, Dalian 116622, China; ^2^Applied Technology College of Dalian Ocean University, Dalian 116300, China

## Abstract

Biocomputing, especially DNA, computing has got great development. It is widely used in information security. In this paper, a novel algorithm of reversible data hiding based on DNA computing is proposed. Inspired by the algorithm of histogram modification, which is a classical algorithm for reversible data hiding, we combine it with DNA computing to realize this algorithm based on biological technology. Compared with previous results, our experimental results have significantly improved the ER (Embedding Rate). Furthermore, some PSNR (peak signal-to-noise ratios) of test images are also improved. Experimental results show that it is suitable for protecting the copyright of cover image in DNA-based information security.

## 1. Introduction

With wide usage of multimedia technologies and excessive spread of Internet, protecting copyright of digital image is attracting a great deal of attention. Data hiding or watermarking technique, which is a major means of protecting copyright, is widely used in digital image and is quite efficient method. Because of their features of parallelism, high-capacity storage, and low energy consumption, DNA computing is used into many fields, such as image encryption, information security, and other applications [1–9].

Reversible data hiding or watermarking and irreversible data hiding are two main technologies for protecting copyright. Reversible data hiding embeds information bits by modifying the host signal but enables the exact (lossless) restoration of the original host signal after extracting the embedded information. Irreversible data hiding cannot enable the exact (lossless) restoration of the original host signal after extracting the embedded information. The former is widely used in the medical image, military information, and other applications with high security requirement. Fridrich et al. proposed a reversible image watermarking algorithm [10]. They formulated two general methodologies for lossless embedding that could be applied to images as well as any other digital objects. Xuan et al. proposed a novel distortionless image data hiding algorithm based on integer wavelet transform [11]. The proposed algorithm could invert the stegoimage into the original image without any distortion after the hidden data were extracted. Tian presented a novel reversible data-embedding method for digital images [12]. The method explored the redundancy in digital images to achieve very high embedding capacity and keep low distortion. The histogram modification method was used into reversible data hiding by Ni et al. [13]. This algorithm utilized the zero or the minimum points of the histogram of an image and slightly modifies the pixel grayscale values to embed data into the image. It was proved analytically and shown experimentally that the peak signal-to-noise ratio (PSNR) of the marked image generated by this method versus the original image was guaranteed to be above 48 dB. According to the above four main methods, a number of works based on reversible data hiding are recently proposed [14–18].

A DNA sequence consists of four different bases, namely, A (adenine), C (cytosine), G (guanine), and T (thymine). Base pairs, which form between specific nucleobases (also termed nitrogenous bases), are the building blocks of the DNA double helix and contribute to the folded structure of both DNA and RNA, namely, A with T and C with G [19]. Adleman used molecular biology to solve an instance of the directed Hamiltonian path problem [1]. There was a stirring of interest in DNA computing after his research. Lipton employed DNA computing to solve the famous “SAT” problem of computer science [2]. Ouyang et al. solved the maximal clique problem by DNA computing [3]. Braich et al. proposed a solution of a 20-variable 3-SAT problem on a DNA computer [4]. Qian et al. used DNA strand displacement to simulate four points of neural network computation [5]. Chang et al. employed DNA computing to achieve factoring integers and break RSA public encryption algorithm [6]. Shoshani et al. proposed a molecular cryptosystem for images by DNA computing [7]. Babaei proposed a reliable data encryption algorithm, One-Time-Pad algorithm (OTP), which is theoretically unbreakable [8]. Liu et al. addressed a RGB color image encryption method based on logistic chaotic sequence and DNA computation [9]. Recently, Chang et al. proposed the new applications of DNA computing, quadratic congruence, and factoring integers. In [20], the authors used an adapted multiobjective version of the differential evolution metaheuristics to design reliable DNA libraries. Jiao et al. proposed an unsupervised spectral matching classifier to perform the task of clustering different ground objects in specific spectral DNA feature encoding subspaces [21]. Zhou et al. designed a new tile self-assembly model to solve the maximum matching problem. In [22], the author proposed a generic delay gate that could be interfaced with virtually any DNA system and presented a theoretical proof of concept of its applicability.

In this paper, we propose a novel algorithm of reversible data hiding based on DNA computing. The algorithm of histogram modification proposed by Ni et al. that is a classical algorithm for reversible data hiding [13]. Inspired by this algorithm, DNA computing is used to realize this algorithm. Compared with previous results, our experimental results have significantly improved the ER (Embedding Rate). Furthermore, some PSNR (peak signal-to-noise ratio) of test images are also improved. Experimental results show that it is suitable for protecting the copyright of cover image in DNA-based information security.

The paper is organized as follows. In the next section, the related works are described in detail. In Section 3, the proposed algorithm is described in detail, and performance analyses and simulation results are reported. Finally, conclusions are drawn in Section 4.

## 2. Related Works

### 2.1. Histogram Modification

The histogram modification was proposed in Ni et al.'s paper [13]. In his method, a zero point (zp for short) and a peak point (pp for short) of the histogram are firstly found. In order to shift the histogram, the grayscale value of pixels between zp and pp is incremented by “1.” It could leave one grayscale value empty and embed watermarking in the pp. For the process of extracting watermarking, bit “1” is extracted from the pixel with the value pp + 1, and bit “0” is extracted from the pixel with the value pp. For others histogram, zp, pp], the pixel value is subtracted by 1. Completing the reverse process, the original image can be recovered without any distortion.

### 2.2. DNA Coding

DNA coding is the key step for DNA computing [23–25]. For the binary bit, 0 with 1 is complementary. So 00 with 11 are complementary, and 01 with 10 are also complementary. In this paper, we consider A = 00, T = 11, C = 01, and G = 10 to encode binary message to DNA sequences. There are eight DNA coding methods to convert binary message to DNA sequences that are stated in Table 1. Here, we use the first DNA coding method.

Mao et al. reported a one-dimensional algorithmic self-assembly of DNA triple-crossover molecules that could be used to execute four steps of a logical (cumulative XOR) operation on a string of binary bits [26]. Rothemund et al. reported a molecular realization, using two-dimensional self-assembly of DNA tiles, of a cellular automaton whose updated rule computes the binary function XOR [27]. Frezza et al. reported the design and functional characterization of a complete set of modular DNA-based Boolean logic gates (AND, OR, and AND-NOT) and further demonstrated their wiring into a three-level circuit that exhibited Boolean XOR (exclusive OR) function [28]. Recently, Shi et al. constructed DNA molecular systems based on DNA strand displacement performing computation of logic gates, including AND, OR, and XOR logic gates [29]. These works show that DNA computing can be used to realize the XOR operation. In this paper, we define the XOR results from two DNA bases. The results are listed in Table 2.

## 3. The Algorithm of Reversible Data Hiding Based on DNA Computing

### 3.1. Embedding Watermarking

In this paper, some standard test images are used to test the effect of proposed algorithm. The cover image firstly is encoded into DNA sequences based on the first row in Table 1. In Ni's algorithm, we should find a zero point and a peak point. In our algorithm, each pixel of cover image is encoded into four bases. Summing up the number of last base for each DNA sequence, the base with max number is found out. According to the max number, the length of watermarking is determined. The pseudocode of Ni's algorithm is briefly stated as follows [14]:

1. Generate its histogram *H*(*x*).
2. In the histogram *H*(*x*), find the peak point *h*(*p*) and zero point *h*(*z*).
3. If the zero point *h*(*z*) > 0, recode the coordinate (*i*, *j*) of those pixels and the pixel grayscale value *b* as overhead bookkeeping information. Then set *h*(*z*) = 0.
4. Without loss of generality, assume *p* < *z*. Move the whole part of the histogram *H*(*x*) with *x* ∈ (*p*, *z*) to the right by 1 unit. This means that all the pixel grayscale values are added by 1.
5. Scan the image, when meeting the pixel, check the to-be-embedded hit. If the to-be-embedded bit is “1,” the pixel grayscale value is changed to *p* + 1. If the bit is “0,” the pixel value remains *p*.

**Figure 1:**
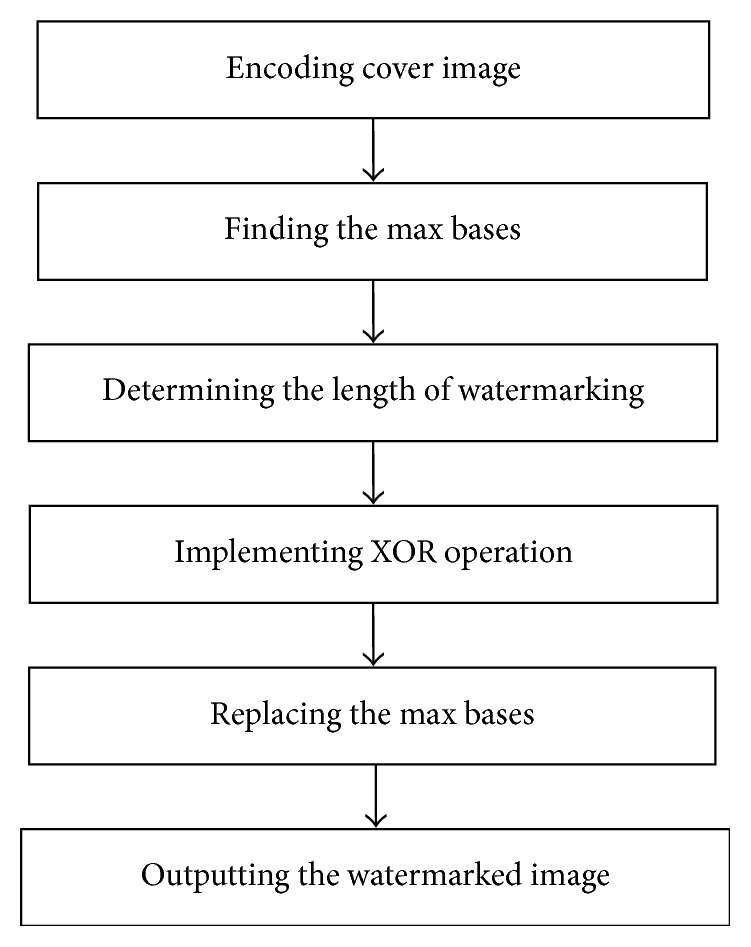
The flowchart of embedding watermarking.

Using the same rule, we encode the watermarking into DNA sequence. Then embedding watermarking combines the max base with the watermarking base by using XOR operation from Table 2. The max bases of cove image are replaced with the results of XOR operation. The detailed of embedding watermarking is illustrated in Figure 1.

Note that the proposed algorithm should reserve one max base and the location of all bases in the cover image. They are corresponding to the pp and zp for the histogram modification and used to recover original image without any restoration. The proposed algorithm does not increase the size of image, so the space complexity is *O*(*n*). The proposed algorithm mainly includes five steps, and their time complexity is *O*(*n*) (encoding cover image), *O*(*n*) (finding the max bases), *O*(1) (determining the length of watermarking), *O*(1) (implementing XOR operation), and *O*(1) (replacing the max bases). So the time complexity of proposed algorithm is *O*(*n*).

### 3.2. Extracting Watermarking

The extracting watermarking process is similar to that of embedding procedure in the reversed order. It can be briefly stated as follows.

**Step 1 .**

Obtain the max base and the location and encode the watermarked image.

**Step 2 .**

According to Table 2, extract watermarking by using XOR operation.

**Step 3 .**

Replace the last bases of the watermarked image with the XOR results of Step 2.

**Step 4 .**

Decode the watermarking and the image after extracting watermarking.

**Step 5 .**

Output the watermarking and image.

### 3.3. Performance Analyses and Simulation

In this chapter, performance analyses and simulation of proposed algorithm are described in detail.

#### 3.3.1. PSNR and ER

The PSNR (peak signal-to-noise ratio) and ER (Embedding Rate) can be used to evaluate the effect of algorithm for image watermarking. The PSNR is defined as

(1) $$\begin{array}{c} \text{PSNR}=10\times \log_{10}\frac{255^{2}}{\text{MSE}}, \end{array}$$

where MSE = (1/*M* × *N*)∑_*i*=1_^*M*×*N*^(*X*_*i*_ − *X*_*i*_′)^2^; *M* and *N* are image width and length, respectively; and *X*_*i*_ and *X*_*i*_′ are pixel value of the original image and watermarked image, respectively. For the histogram modification algorithm, the difference between *X*_*i*_ and *X*_*i*_′ is equal to 1 in the worst case; namely, all the pixels are changed. So MSE = (1/*M* × *N*)∑_*i*=1_^*M*×*N*^(1)^2^ = 1, and PSNR = 48.13 dB. The ER is the Bits Per Pixel and defined as

(2) $$\begin{array}{c} \text{ER}=\frac{\text{Nu}{\text{m}}_{\text{wk}}}{\text{Nu}{\text{m}}_{\text{pixels}}}\;\;\left(\;\text{bpp}\;\right), \end{array}$$

where Num_wk_ denotes the number of embedding watermarking and Num_pixels_ denotes the number of pixels of cover image. For the histogram modification algorithm, all the pixels of cover image embedded one-bit watermarking in the best case, so the maximum of ER is equal to 1 bpp.

#### 3.3.2. Simulation and Experiment

In this section, four different standard images with size 512*∗*512 are used to test the effect of proposed algorithm, respectively, Lena, Airplane, Baboon, Boat, House, and Tiffany.

**Figure 2:**
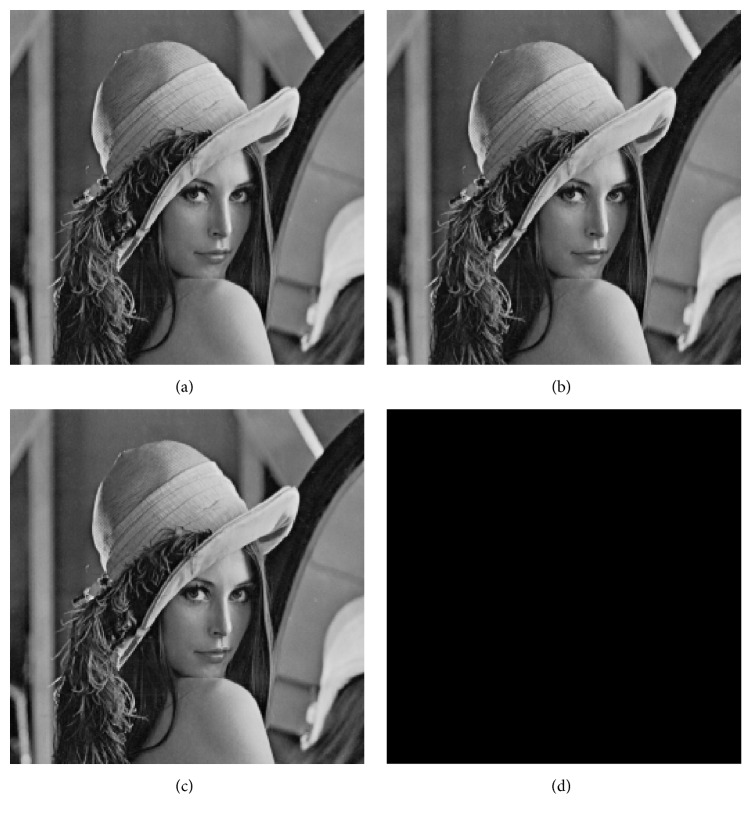
(a) Lena; (b) watermarked image; (c) after extracting watermarking; (d) difference image.

**Figure 3:**
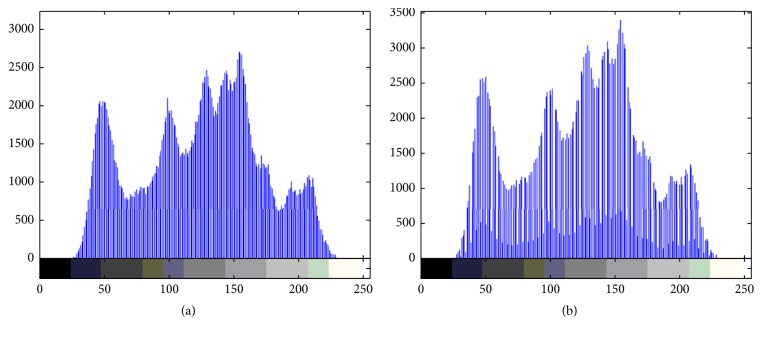
(a) Histogram of the cover image Lena; (b) histogram of the watermarked image Lena.

Figure 2 shows the effect of proposed algorithm.  Figure 2(a) is the original cover image, Figure 2(b) is the watermarked image, Figure 2(c) is the recovered imager after extracting watermarking, and Figure 2(d) is the difference image between Figures 2(a) and 2(b). Figure 2(d) is an all-black figure, so the test image can be reversibly recovered by our algorithm.  Figure 3 shows the changes of histogram of Lena.  Figure 3(a) is the histogram of cover image, and Figure 3(b) is the histogram of watermarked image.

**Figure 4:**
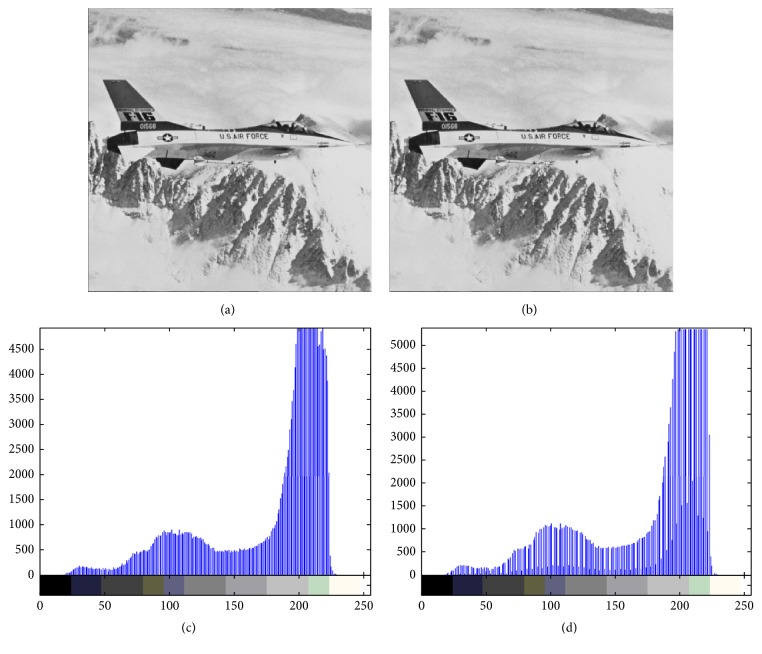
(a) Airplane; (b) watermarked image; (c) histogram of (a); (d) histogram of (b).

**Figure 5:**
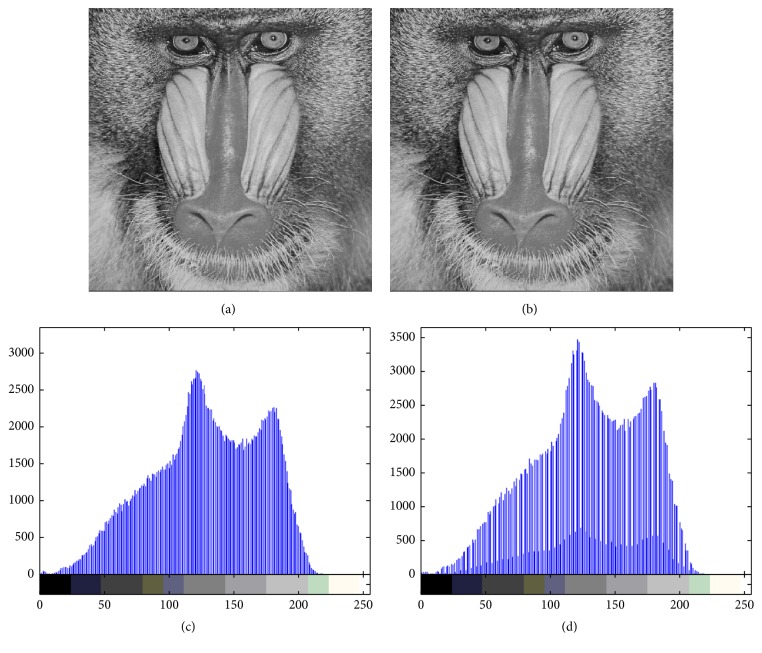
(a) Baboon; (b) watermarked image; (c) histogram of (a); (d) histogram of (b).

**Figure 6:**
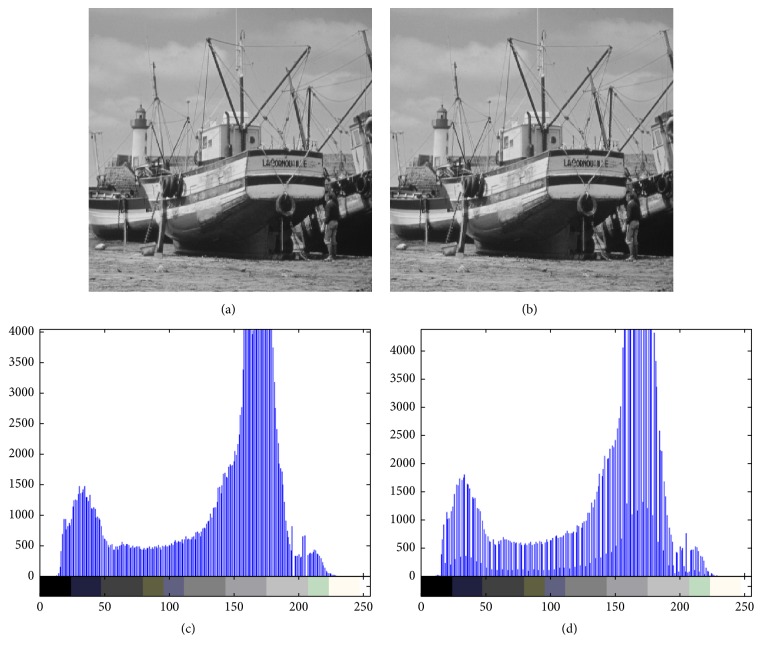
(a) Boat; (b) watermarked image; (c) histogram of (a); (d) histogram of (b).

**Figure 7:**
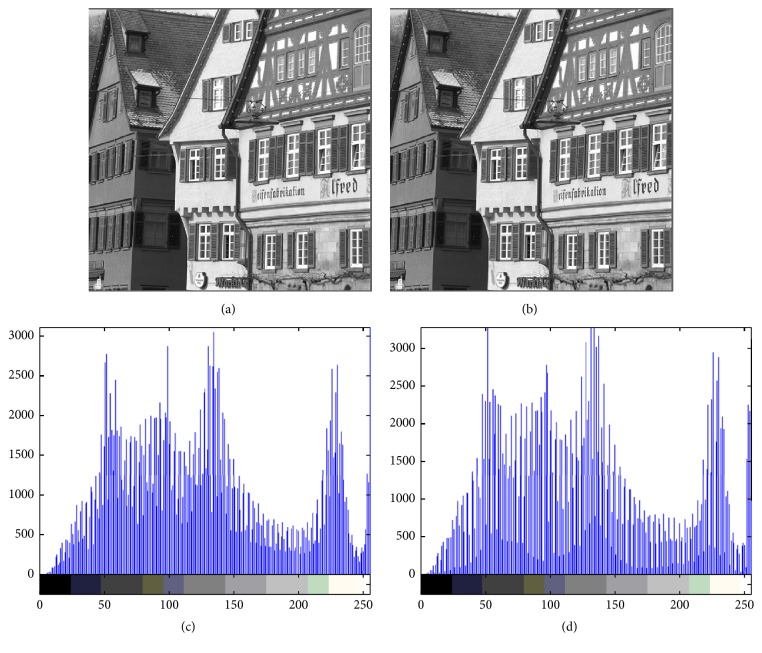
(a) House; (b) watermarked image; (c) histogram of (a); (d) histogram of (b).

**Figure 8:**
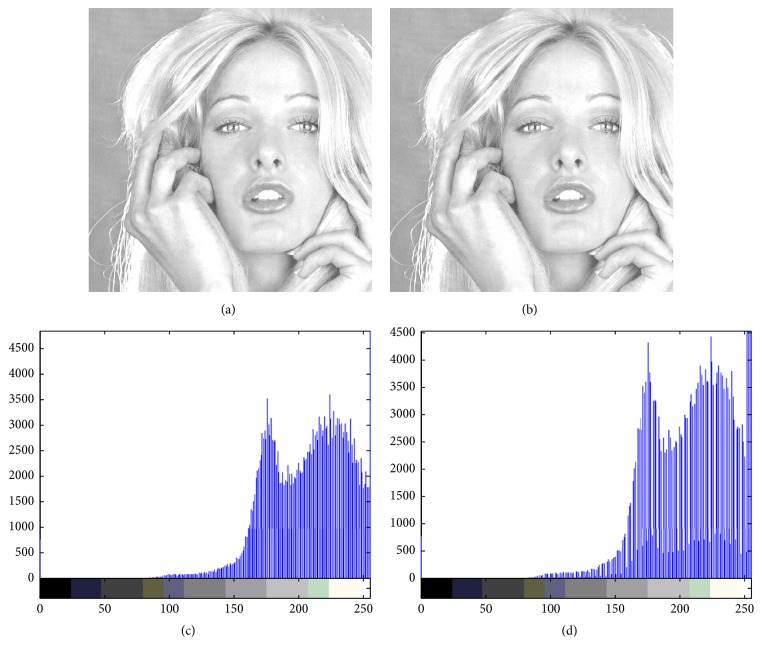
(a) Tiffany; (b) watermarked image; (c) histogram of (a); (d) histogram of (b).

Figures 4–8 are other test cover images, Airplane, Baboon, Boat, House, and Tiffany, respectively. Each subimage (a) is a cover image. Each subimage (b) is the watermarked image. Each subimage (c) is the histogram of (a) and (d) is the histogram of (b).

Table 3 reports the results of Ni's algorithm [13].  Table 4 lists the results of our algorithm. Compared with these results, our algorithm significantly improves the number of watermarking bits and ER. Some PSNR of test images are also improved, except for Baboon and Boat. But small differences do not influence greatly the quality of watermarked image. In Ni's algorithm, the watermarking is embedded in peak point, which is a pixel of cover image. Its value is from range of 0 to 255. In proposed algorithm, we make the max base as the peak point, which is from range of A to T, namely, A, C, G, and T. So the proposed algorithm could increase the number of peak points and ER.

## 4. Conclusions

In this work, we combine image watermarking with DNA computing that proposes a novel algorithm for reversible data hiding. We firstly introduce the method of histogram modification, which is a famous algorithm and proposed by Ni et al. [13], and the background of DNA computing. Combined with the merits of DNA computing, we realize reversible data hiding based on biological technology. Compared with previous results, our experimental results have significantly improved the ER. Furthermore, some PSNR of test images are also improved. Experimental results show that it is suitable for protecting the copyright of cover image in DNA-based information security. In the future work, we will attempt to use DNA computing and quantum computing to hide watermarking.

## Figures and Tables

**Table 1:** DNA coding list.

	1	2	3	4	5	6	7	8
A	00	00	01	01	10	10	11	11
T	11	11	10	10	01	01	00	00
C	01	10	00	11	00	11	01	10
G	10	01	11	00	11	00	10	01

**Table 2:** XOR results.

XOR	A	C	G	T
A	A	C	G	T
C	C	A	T	G
G	G	T	A	C
T	T	G	C	A

**Table 3:** The results of Ni's algorithm [13].

Images	PSNR (dB)	Watermarking (bits)	ER (bpp)
Lena	48.2	5460	0.021
Airplane	48.3	16171	0.060
Baboon	48.2	5421	0.021
Boat	48.2	7301	0.028
House	48.3	14310	0.055
Tiffany	48.2	8782	0.034

**Table 4:** The results of our algorithm.

Images	PSNR (dB)	Watermarking (bits)	ER (bpp)
Lena	48.3	131656	0.502
Airplane	51.5	133764	0.510
Baboon	47.8	131654	0.502
Boat	48.0	138352	0.528
House	48.4	141496	0.540
Tiffany	49.1	157174	0.600
